# Metformin modulates oxidative stress via activation of AMPK/NF-κB signaling in Trisomy 21 fibroblasts: an *in vitro* study

**DOI:** 10.3389/fmolb.2025.1577044

**Published:** 2025-06-09

**Authors:** Angelika Buczyńska, Piotr Malinowski, Arkadiusz Żbikowski, Adam Jacek Krętowski, Monika Zbucka-Krętowska

**Affiliations:** ^1^ Clinical Research Centre, Medical University of Bialystok, Bialystok, Poland; ^2^ Department of Gynecological Endocrinology and Adolescent Gynecology, Medical University of Białystok, Białystok, Poland; ^3^ Department of Endocrinology, Diabetology and Internal Medicine, Medical University of Bialystok, Bialystok, Poland

**Keywords:** Trisomy 21, oxidative stress, metformin, Transcription factors, antioxidant capacity

## Abstract

**Introduction:**

Oxidative stress and impaired antioxidant defenses are key contributors to cellular dysfunction in Trisomy 21 (T21), highlighting the need for targeted therapeutic strategies. This study explores the modulatory effects of metformin on oxidative stress and antioxidant capacity in T21.

**Methods:**

An *in vitro* model was employed using human fibroblast cells with T21 (CCL-54 – Detroit 532 and Detroit 539 – CCL-84; ATCC) alongside normal fibroblasts as a control group (PCS-201-012; ATCC). These cells were treated with varying doses of metformin (10 μM, 30 μM, and 50 μM) for 48 h to assess its pleiotropic protective effects and their impact on oxidative-metabolic cellular profiles.

**Results:**

Our results demonstrate that metformin treatment significantly reduced total oxidative capacity (TOC) and levels of oxidative DNA/RNA damage products in T21 cell lines (CCL-84 and CCL-54). Additionally, metformin markedly increased total antioxidant capacity (TAC) in these fibroblasts. Furthermore, metformin influenced key signaling pathways, as evidenced by increased levels of nuclear factor kappa B (NF-κB) and enhanced activity of protein kinase AMP-activated alpha 1 (PRKAA1) and AMP-activated protein kinase (AMPK) in T21 cell lines.

**Conclusions:**

These findings highlight metformin’s significant role in modulating oxidative stress and inflammation- related mechanisms in T21. Given the growing interest in managing oxidative stress during pregnancies affected by T21, this study presents potential clinical implications for therapeutic intervention.

## 1 Introduction

Trisomy 21 (T21), manifested as the Down syndrome (DS) phenotype, remains one of the most prevalent chromosomal disorders, affecting approximately 1 in 700 live births worldwide (Sherman et al., 2007; Ghosh et al., 2011). Its incidence increases with maternal age, posing a significant challenge in both maternal and neonatal health (Aversa et al., 2018; Al-Nbaheen, 2018; Crawford and Dearmun, 2016). Despite advancements in prenatal diagnostics and postnatal care, T21 continues to be associated with a high prevalence of congenital abnormalities and developmental disorders (Hasina and Wang, 2022). The underlying mechanisms of these abnormalities, particularly those linked to cellular metabolic dysfunctions, are not fully understood, thus effective therapeutic targets have yet to be clearly defined (Glasson et al., 2002; Silverman, 2007; Bartesaghi et al., 2011). Nevertheless, oxidative stress is a well-documented hallmark ofT21 (Gimeno et al., 2014; Slonim et al., 2009), arising from the overexpression of genes located on human chromosome 21 (HSA21), such as *superoxide dismutase 1 (SOD1), amyloid beta precursor protein (APP), and BTB domain and CNC homolog 1 (BACH1)*, which contribute to redox imbalance and mitochondrial dysfunction (Perluigi et al., 2020; Patterson, 2007; Buczyńska et al., 2023). Thus, overexpression of specific genes located on chromosome 21 plays a central role in promoting redox imbalance and mitochondrial dysfunction in T21. One key contributor is SOD1, which catalyzes the dismutation of superoxide radicals to hydrogen peroxide. In T21, the triplication of SOD1 results in excessive H_2_O_2_ production that overwhelms detoxification systems, promoting oxidative stress. Additionally, APP (amyloid precursor protein) contributes to mitochondrial dysfunction by increasing mitochondrial fragmentation and reactive oxygen species (ROS) production through Aβ-related mechanisms. BACH1, a transcriptional repressor also encoded on chromosome 21, interferes with the expression of genes involved in antioxidant responses, such as HO-1 (heme oxygenase 1), further weakening cellular defense systems. Together, these gene dosage effects establish a pro-oxidative cellular environment that disrupts energy homeostasis and cellular signaling pathways, including AMP-activated protein kinase (AMPK) and nuclear factor kappa beta (NF-κB), ultimately contributing to the metabolic and developmental disturbances observed in T21. Moreover, a genome-wide analysis of differentially expressed cell-free microRNAs in the amniotic fluid of fetuses with T21 identified dysregulated genes primarily involved in AMPK signaling pathways (Zhou et al., 2023). [NO_PRINTED_FORM] (Grzeszczak et al., 2023; Toboła-Wróbel et al., 2020; Perluigi et al., 2011; Duhig et al., 2016), Excessive ROS production in T21 disrupts developmental processes, exacerbating metabolic imbalance. Astrocytes, the most abundant glial cells in the brain, are particularly susceptible to oxidative stress, and their apoptosis is implicated in the neurodevelopmental defects associated with T21. Emerging evidence indicates that oxidative stress manifests early in T21 pregnancies, suggesting its relevance as a potential therapeutic target for mitigating downstream metabolic and neurodevelopmental abnormalities (Perluigi et al., 2011; Ponroy Bally and Murai, 2021; Perluigi and Butterfield, 2012; Dierssen et al., 2020; Cai et al., 2024). Therefore, Valenti et al. reported that human fetal skin fibroblasts with T21 exhibited a deficiency in mitochondrial respiratory chain complex I, associated with reduced cAMP-dependent phosphorylation of its 18-kDa subunit. This impairment resulted from decreased AMPK activity, which was linked to lower basal cAMP levels. Treatment with dibutyryl-cAMP (db-cAMP), a membrane-permeable cAMP analogue, restored AMPK activity, thereby rescuing both the phosphorylation status and enzymatic function of complex I. Conversely, inhibition of AMPK using H89 abolished these effects. Importantly, a threefold increase in ROS, particularly superoxide anion, was detected, predominantly originating from DS-HSF mitochondria (Valenti et al., 2011). In the context of T21 fibroblasts, this signaling cascade restores mitochondrial respiratory chain complex I activity, enhances ATP production, and reduces ROS generation by improving mitochondrial efficiency. The activation of AMPK through this pathway counteracts the reduced basal cAMP levels observed in T21 cells. By re-establishing AMPK activity, db-cAMP effectively mitigates oxidative stress and promotes energy homeostasis, offering proof-of-concept for AMPK-centered therapeutic strategies. These findings provide a mechanistic rationale for exploring pharmacological agents like metformin that act as direct or indirect AMPK activators. Dibutyryl-cAMP, despite showing promising results in the modulation of the AMPK pathway, has primarily served as a basis for further research, as it has not been applied in animal models or clinical studies involving humans.

Metformin, a widely used drug in the treatment of type 2 diabetes, has gained attention as a potential therapeutic approach for conditions associated with DS. Due to its anti-inflammatory properties and ability to counteract oxidative stress, metformin emerges as a promising candidate for mitigating the metabolic and neurodevelopmental abnormalities observed in T21. Its mechanism of action also involves the activation of the AMPK pathway, leading to enhanced antioxidant defenses through modulation of *BACH1*, as well as the regulation of inflammatory pathways such as NF-κB (Chiang et al., 2017; Picone et al., 2015; Kefas et al., 2004; Kanigur Sultuybek et al., 2019; Fischhuber et al., 2020). PRKAA1, a catalytic subunit of AMPK, plays a critical role in cellular energy sensing and metabolic regulation, and its impaired function in T21 may exacerbate energy deficits and oxidative stress (Yun and Zierath, 2006). By stimulating AMPK, metformin may help restore cellular energy homeostasis, enhance mitochondrial function, and improve redox balance in T21 cells (Yun and Zierath, 2006; Garcia and Shaw, 2017; Herzig and Shaw, 2017; Wang et al., 2019). Given these findings, metformin represents a strong therapeutic candidate to target mitochondrial dysfunction in T21. By inhibiting complex I, metformin modulates mitochondrial respiration, reduces ROS production, and activates AMPK, a central regulator of cellular energy and redox homeostasis. Its excellent safety profile, proven placental transfer, and increasing use in oxidative stress–related conditions such as polycystic ovary syndrome further support its potential therapeutic application in T21 [(Bost et al., 2018; Kurzthaler et al., 2014; Nassif et al., 2024; Cai et al., 2024; Buczyńska et al., 2024; Ugwueze et al., 2020; Lin et al., 2023; Dutta et al., 2023; Buczyńska et al., 2022)]. In addition to its antioxidant properties, metformin has shown potential in protecting against oxidative DNA and RNA damage, which are significantly elevated due to oxidative stress in T21 pregnancies (Al-Nbaheen, 2018; Bartesaghi et al., 2011; Buczyńska et al., 2023; Buczyńska et al., 2021a; Buczyńska et al., 2020; Buczyńska et al., 2021b; Revilla and Martínez-Cué, 2020; Chiang et al., 2017; Picone et al., 2015; Kefas et al., 2004; Kanigur Sultuybek et al., 2019; Fischhuber et al., 2020; Zhu et al., 2013; Yun and Zierath, 2006; Al-Nbaheen, 2018; Bartesaghi et al., 2011; Buczyńska et al., 2023; Chiang et al., 2017; Picone et al., 2015; Kefas et al., 2004; Kanigur Sultuybek et al., 2019; Fischhuber et al., 2020; Yun and Zierath, 2006; Yun and Zierath, 2006; Garcia and Shaw, 2017; Herzig and Shaw, 2017; Wang et al., 2019; Buczyńska et al., 2021a; Buczyńska et al., 2021a; Buczyńska et al., 2020; Buczyńska et al., 2021b; Revilla and Martínez-Cué, 2020; Zhu et al., 2013).

This study aims to evaluate the potential of metformin as a therapeutic agent for T21 by assessing its ability to modulate oxidative stress and regulate key signaling pathways, including AMPK and NF-κB. Given that oxidative stress plays a crucial role in T21-related metabolic and neurodevelopmental impairments, metformin’s ability to enhance endogenous antioxidant defenses and restore AMPK/NF-κB activity may provide protective effects at both the cellular and systemic levels. By targeting these dysregulated pathways, this study lays the groundwork for developing novel prenatal and postnatal therapeutic strategies aimed at mitigating T21-associated comorbidities.

## 2 Materials and methods

### 2.1 Cell culture and handling

The fibroblasts used in this study were commercially available human fibroblast cell lines obtained from the American Type Culture Collection (ATCC). The T21 fibroblast lines included Detroit 532 (CCL-54, neonatal male) and Detroit 539 (CCL-84, pediatric female), while the control fibroblast line (PCS-201-012) was derived from a healthy donor. Before conducting the experiments, the fibroblast lines were 6 times expanded and passaged to maintain cellular integrity and avoid genetic drift associated with prolonged culture. The T21 genotype of the fibroblast lines was confirmed by ATCC through karyotyping and molecular validation prior to purchase. Additionally, routine morphological assessment and growth characteristics were monitored in our laboratory to ensure consistency with previously reported T21 fibroblast phenotypes. Although commercially available, these cell lines are widely recognized in research on T21 and provide a reliable and standardized model for studying oxidative stress and metabolic dysregulation in those cells. Cells were cultured in Dulbecco’s Modified Eagle Medium (DMEM; Gibco) supplemented with 10% fetal bovine serum (FBS; Sigma-Aldrich), 1% penicillin/streptomycin (Gibco), and 2 mM L-glutamine (Gibco). Fibroblast cell lines were first cultured and expanded in T-75 flasks (75 cm^2^ surface area). Cultures were maintained at 37°C in a humidified incubator with 5% CO_2_, and the medium was replaced every 24 h. The 48-h incubation period was selected based on prior studies in human fibroblasts showing that metformin induces measurable effects on AMPK signaling, redox balance, and NFκB activity within this timeframe, while preserving high cell viability (Kazkayasi and Denizalti, 2023; Moiseeva et al., 2013; Sainio et al., 2019; Rajh et al., 2016; Algire et al., 2012; Martin-Montalvo et al., 2013).

### 2.2 Cell density and metformin treatment

Cells were seeded at a density of 1.5 × 10^5^ cells/well in 6-well plates and allowed to adhere for 24 h before treatment. Metformin hydrochloride (Sigma-Aldrich, catalog no. D150959) was freshly dissolved in sterile phosphate-buffered saline (PBS, pH 7.4), and the working solution was sterilized using a 0.22 μm syringe-driven nanofilter (Millex® GV, Millipore) to eliminate any potential microbial contaminants. Final concentrations of 10 μM, 30 μM, and 50 μM were prepared and added directly to the culture medium. Control wells received an equivalent volume of filtered PBS as a vehicle control. Following treatment, cells were incubated for an additional 48 h under standard conditions. The selected metformin doses were based on a review of the available literature, which indicates that metformin plasma concentrations in patients receiving therapeutic doses (1,500 mg/day) do not exceed 30 μM (Buczyńska et al., 2022; Buczyńska et al., 2021a; Buczyńska et al., 2020). The 48-h incubation period was chosen based on published studies demonstrating that metformin elicits measurable effects on AMPK signaling, mitochondrial function, and redox homeostasis in human cell cultures within this time frame. This duration offers a balance between sufficient pathway activation and cell viability, while minimizing confounding effects from prolonged exposure. To verify the safety and non-toxicity of the applied doses, cell viability was assessed using the MTT assay (CyQUANT™ MTT Cell Viability Assay Kit, Invitrogen, Cat. No. V13154), following the manufacturer’s protocol. Optical density was measured at 570 nm using a microplate reader (BioTek Synergy H1).

### 2.3 Cell lysis and protein extraction

Following metformin treatment, cells were washed twice with cold PBS and lysed using RIPA buffer (Thermo Fisher) supplemented with a 1× protease and phosphatase inhibitor cocktail (Roche). Lysates were incubated on ice for 30 min with periodic vortexing and then centrifuged at 14,000 × g for 15 min at 4°C. The supernatants were collected and stored at −80°C for subsequent analysis.

### 2.4 Signaling pathway and oxidative stress analysis

The oxidation-reduction colorimetric technique was used to determine levels of total oxidant capacity (TOC) and total antioxidant capacity (TAC). TOC was evaluated using a commercial photometric immunodiagnostic test (PerOx TOS/TOC, KC5100, 64625 Bensheim, Germany). TAC was assessed using a commercial photometric test (ImAnOx TAS/TAC, KC5200, 64625 Bensheim, Germany). Oxidative DNA/RNA damage products (OSDP DNA/RNA) were measured using the DNA/RNA Oxidative Damage (High Sensitivity) ELISA Kit (Cayman Chemicals, Ann Arbor, Michigan, MI, USA, 589320), which allows simultaneous detection of OSDP DNA/RNA, such as 8-hydroxyguanosine (8-OHG), 8-hydroxy-2′-deoxyguanosine (8-OHdG), and 8-hydroxyguanine. AMPK activity was measured using an enzyme immunoassay (ab151280, ABCAM). The concentrations of NFκB and the PRPKAa1 were determined using an enzyme-linked immunosorbent assay (ELISA kits; Cloud-Clone Corp., Wuhan, China; SEB824Hu; SEA679Hu, respectively), according to the manufacturer’s instructions in 5 repetitions of determinations in cell lysates.

### 2.5 Statistical analysis

Statistical analyses were carried out using GraphPad Prism version 9.0 software (GraphPad Software, Inc., San Diego, CA, USA). The Shapiro-Wilk test indicated a non-normal distribution of the data. Therefore, the Mann-Whitney U test was applied for comparisons between independent groups, and the Wilcoxon signed-rank test was used for comparisons before and after treatment within the same cell line. The p-value <0.05 was considered statistically significant.

## 3 Results

### 3.1 Cell profile characterization

**FIGURE 1:**
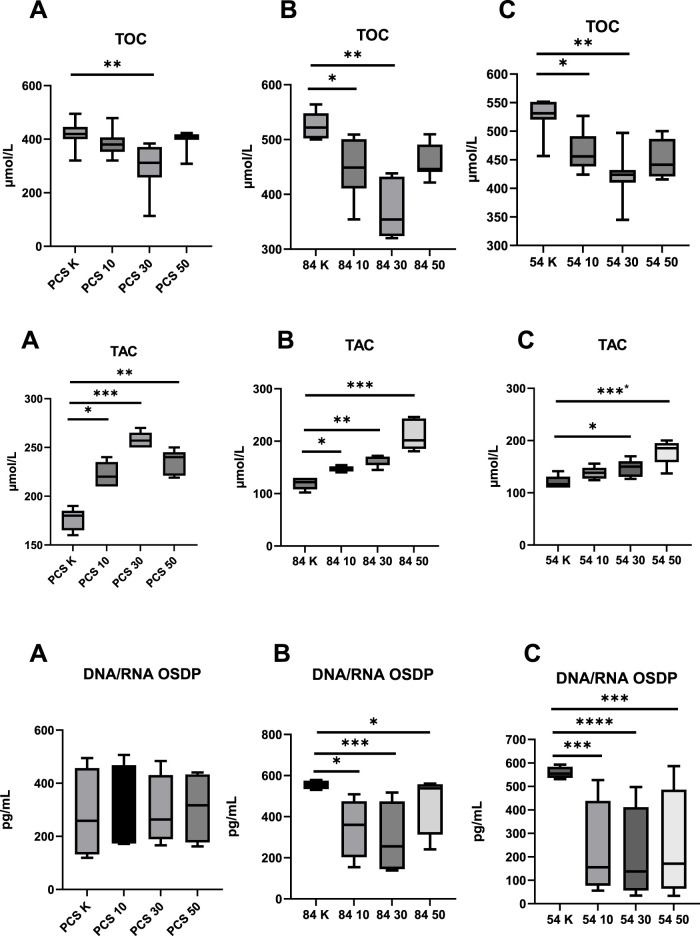
Evaluation of Parameters Assessing Oxidative Status before and after metformin treatment (10–10 µM dose; 30–30 µM dose; 50–50 µM dose; K – reference baseline time point), compared to the reference cell line. TOC, total oxidative capacity; TAC, total antioxidant capacity; DNA/RNA OSDP, DNA/RNA oxidative stress damage products; **(A)** PCS–PCS-201-012; **(B)** 84 – CCL-84; **(C)** 54 – CCL-54. Significant differences compared to the reference cell line are indicated as follows: *p < 0.05, **p < 0.01, ***p < 0.001.; ****p < 0.000. Statistical analysis was performed using [GraphPad Software, Inc., San Diego, CA, USA].

**FIGURE 2:**
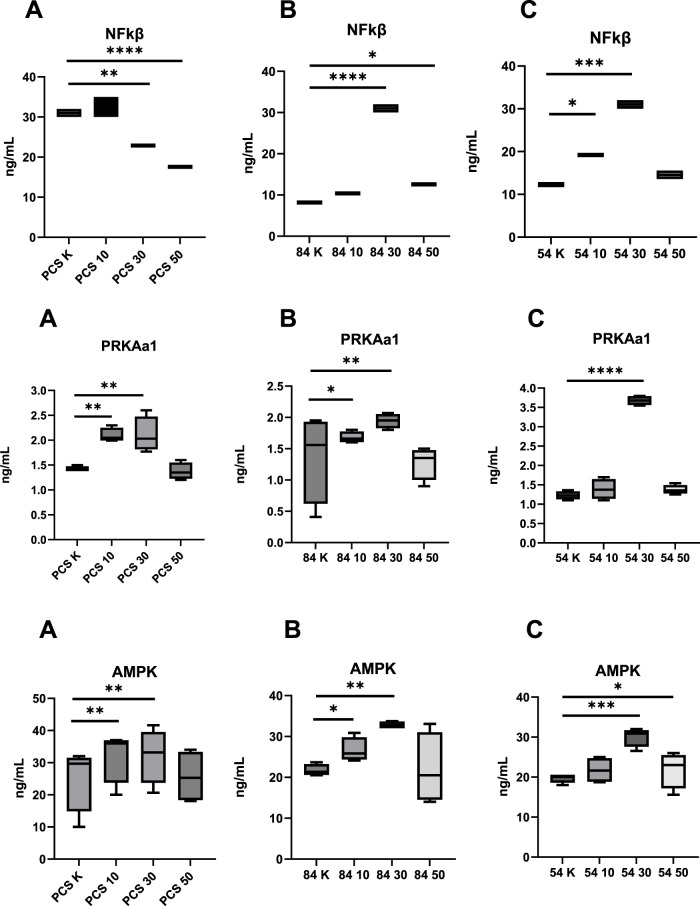
Assessment of the Concentration of Modulating Factors Affecting Oxidative Capacity Following Metformin Stimulation before and after metformin treatment (10–10 µM dose; 30–30 µM dose; 50–50 µM dose; K, reference baseline time point), compared to the reference cell line. NF-κB, nuclear factor kappa B; AMPK, AMP-activated protein kinase; PRKAA1, catalytic subunit alpha-1 of AMP-activated protein kinase; **(A)** PCS–PCS-201-012; **(B)** 84 – CCL-84; **(C)** 54 – CCL-54. Significant differences compared to the reference cell line are indicated as follows: *p < 0.05, **p < 0.01, ***p < 0.001.; ****p < 0.000. Statistical analysis was performed using [GraphPad Software, Inc., San Diego, CA, USA.

At baseline, the T21 cell lines (CCL-84 and CCL-54) exhibited increased TOC (p = 0.041; p = 0.034) and DNA/RNA OSDP (p = 0.021; p = 0.036), accompanied by reduced TAC (p = 0.011; p = 0.009) when compared to the control group (PCS-201-012). Furthermore, levels of NF-κB and PRKAA1 were elevated (p = 0.027; p = 0.049) in the control group (PCS-201-012) compared to the T21 cell lines (CCL-84 and CCL-54). No significant differences in AMPK concentration were observed among the studied cell lines (all p > 0.05) (Figures 1, 2).

### 3.2 The impact of metformin on oxidative and antioxidative balance

Oxidative status, measured by TOC and TAC, showed a significant reduction in TOC in the control group using the PCS-201–012 line after the administration of 30 µM metformin (p = 0.024). In the experimental group, which included the CCL-84 and CCL-54 cell lines, a decrease in TOC was observed after the application of 10 μM and 30 µM metformin, compared to pre-intervention levels (p = 0.045; p = 0.036; p = 0.041; p = 0.038) (Figure 1). A 30 µM dose of metformin resulted in a significant reduction in TOC in normal fibroblasts (p < 0.01), while no such effect was observed at 10 μM and 50 µM concentrations. In the T21 cell lines, metformin concentrations of 10 μM and 30 µM also reduced TOC.

Additionally, an increase in TAC was observed after metformin administration in the normal fibroblast model (10 μM; p = 0.043; 30 μM; p < 0.001; 50 μM; p = 0.013) as well as in the T21 cell lines (CCL-84–10 μM; p = 0.042; 30 μM; p = 0.014; 50 μM; p < 0.0001; CCL-54–10 μM; p < 0.042; 30 μM; p < 0.0001) (Figure 1). Metformin did not affect DNA OSDP levels in normal fibroblasts (p > 0.05), whereas in T21 cell lines, metformin caused a reduction in DNA OSDP levels at all tested concentrations.

An increase in TAC was observed after metformin administration at concentrations of 10 μM, 30 μM, and 50 µM in the control group (p = 0.046; p = 0.001; p = 0.026). Similar results were observed in the experimental groups, where the administration of metformin at 10 μM, 30 μM, and 50 µM in the CCL-84 line (p = 0.047, p = 0.011, p < 0.001) and at 30 μM and 50 µM in the CCL-54 line (p = 0.044, p < 0.001) contributed to an increase in TAC (Figure 1).

In T21 cells, the concentration of DNA/RNA oxidative stress damage products (OSDP) decreased after metformin treatment at doses of 10 μM, 30 μM, and 50 µM (CCL-84 - p = 0.04; p < 0.001; p = 0.04; CCL-54 - p < 0.001; p < 0.0001; p < 0.001). No changes in DNA/RNA OSDP levels were observed in the control group with the PCS-201–012 line after metformin treatment (Figure 1).

### 3.3 Impact of metformin on NF-κB, PRKAA1, and AMPK expression

In further analysis, changes in the concentrations of NF-κB, PRKAA1, and AMPK were examined. NF-κB levels decreased in the control group (PCS-201–012 line) after the administration of metformin at doses of 30 µM (p = 0.02) and 50 µM (p < 0.0001). In the experimental group with the CCL-84 line, an increase in NF-κB levels was observed after metformin treatment at doses of 30 µM (p < 0.0001) and 50 µM (p = 0.042). In the CCL-54 line, NF-κB levels increased after the administration of 10 µM (p = 0.047) and 30 µM (p < 0.001) metformin compared to baseline values (Figure 2).

PRKAA1 levels in the control group (PCS-201–012 line) increased after the administration of metformin at doses of 10 µM (p = 0.018) and 30 µM (p = 0.027) compared to pre-intervention values. In the experimental group with the CCL-84 line, PRKAA1 levels increased after metformin treatment at doses of 10 µM (p = 0.038) and 30 µM (p < 0.001). In the CCL-54 line, an increase in PRKAA1 levels was observed after metformin administration at 30 µM (p < 0.0001) (Figure 2).

AMPK activity in the control group increased after metformin administration at doses of 10 µM (p = 0.031) and 30 µM (p = 0.024). In the experimental group with the CCL-84 line, increased AMPK activity was observed after metformin treatment at doses of 10 µM (p = 0.044) and 30 µM (p = 0.026). In the CCL-54 line, AMPK activity increased after the administration of 30 µM (p < 0.001) and 50 µM (p = 0.042) metformin, compared to pre-exposure levels (Figure 2).

### 3.4 Metformin cytotoxicity evaluation

To confirm the non-toxic nature of metformin concentrations used, cell viability was assessed using the MTT assay. The results revealed 100% viability at 10 µM and 30 μM, and 98% viability at 50 µM metformin, indicating no significant cytotoxicity in either control or T21 fibroblasts.

## 4 Discussion

Oxidative stress has been recognized as an early pathogenic factor in T21 pregnancies (Buczyńska et al., 2021a; Žitňanová et al., 2006; Gulesserian et al., 2001; Lim et al., 2014; Izzo et al., 2018; Barone et al., 2018; Busciglio and Yankner, 1995; Skibinska et al., 2021; Kovo et al., 2008; Muchová et al., 2014; Liu et al., 2011; Helguera et al., 2013). Perluigi et al. analyzing amniotic fluid biomarkers, reporting increased levels of protein oxidation, lipid peroxidation, reduced glutathione (GSH), and oxidation of proteins associated with iron homeostasis, lipid metabolism, and inflammatory pathways. These alterations suggest that oxidative stress contributes significantly to the pathogenesis of DS, including developmental abnormalities and Alzheimer’s disease–like neuropathology observed in affected individuals (Perluigi et al., 2011). Subsequent study performed by the Perluigi et al. further supported this concept, showing a nine-fold increase in isoprostanes (IPs)—highly sensitive markers of free radical–induced lipid peroxidation—in the amniotic fluid of T21 pregnancies compared to euploid controls (Perrone et al., 2007). Perluigi et al. (2011) Our previous research also demonstrated a significant increase in DNA/RNA OSDP levels in amniotic fluid samples among pregnancies affected by T21 compared to euploid samples (Buczyńska et al., 2021a). However, maternal plasma measurements did not show significant differences between T21 and euploid pregnancies, indicating that the oxidative imbalance may be localized predominantly to the fetal environment (Buczyńska et al., 2021a; Pietryga et al., 2021). Moreover, oxidative stress appears to persist postnatally. Komatsu et al. quantified 8-hydroxy-2′-deoxyguanosine (8-OHdG), a well-established oxidative DNA damage marker, in the saliva of individuals with DS and confirmed elevated levels, reinforcing the role of oxidative stress as a core feature of the DS phenotype (Komatsu et al., 2013). Collectively, the presented findings contribute to the growing body of evidence implicating oxidative stress as a central pathogenic mechanism in T21 (Buczyńska et al., 2023; Perluigi et al., 2011; Perrone et al., 2007) while simultaneously underscoring the urgent need for the development of targeted pharmacological strategies to mitigate redox imbalance (Guedj et al., 2020; Nikolić et al., 2022). Importantly, these interventions should be considered not only in the postnatal period but also prenatally, as oxidative stress is evident already during fetal development (Revilla and Martínez-Cué, 2020; Reynolds, 2008; Sebastiani et al., 2022; Agarwal et al., 2006). Early therapeutic modulation may offer a valuable opportunity to alter the trajectory of neurodevelopmental and metabolic complications associated with DS with metformin emerging as a promising candidate due to its favorable safety profile and potential neuroprotective and metabolic benefits (Nunomura et al., 2000; Zana et al., 2007).

Our findings demonstrating a significantly elevated TOC, DNA/RNA OSDP and decreased TAC in T21 fibroblasts, compared to euploid controls, confirm the pivotal role of oxidative stress in the pathophysiology of T21 and support the rationale for targeting redox imbalance in pharmacological interventions. The observed reduction in AMPK, and PRKAA1 with NFkB expression in T21 cells further validates the use of this *in vitro* model as a sensitive system for detecting redox fluctuations and assessing metabolic vulnerability. Notably, this model allows for the direct evaluation of metformin as a pleiotropic therapeutic agent capable of modulating multiple pathways implicated in oxidative stress, mitochondrial dysfunction, and impaired energy homeostasis in T21. Therefore, in the control group (PCS-201-012 cell line), a significant reduction in TOC was observed only following treatment with 30 µM metformin. This may reflect a dose-specific threshold effect, wherein 30 µM represents an optimal concentration capable of activating antioxidant pathways without perturbing basal cellular metabolism as was illustrated by AMPK and PRKAA1 activation in all studied cell lines. Lower doses may be insufficient to elicit measurable redox modulation in non-trisomic cells, which possess intact antioxidant defenses and AMPK with NFkB activity, while higher doses may induce counterregulatory mechanisms or transient mitochondrial stress, thereby diminishing the net antioxidative effect. In the experimental group, comprising the CCL-84 and CCL-54 T21 fibroblast lines, a reduction in TOC was observed following treatment with both 10 μM and 30 µM metformin. However, at 50 μM, TOC levels increased relative to the 30 µM dose. This response may reflect a biphasic, dose-dependent effect of metformin, wherein lower concentrations (up to 30 µM) efficiently stimulate mitochondrial function and activate AMPK and NFkB, thereby enhancing cellular energy homeostasis and antioxidant defenses. In contrast, higher concentrations, such as 50 μM, may lead to excessive inhibition of mitochondrial complex I, impairing oxidative phosphorylation and triggering secondary oxidative stress. This paradoxical effect suggests that supraphysiological doses may disrupt cellular signaling and redox balance, highlighting the importance of dose optimization when considering metformin as a therapeutic strategy in T21 (Wang et al., 2019; Wang et al., 2019; Yang et al., 2021). Our findings confirm that metformin effectively reduces oxidative stress and enhances cellular energy homeostasis through AMPK and PRKAA1 pathway activation in T21 fibroblastsThus, our study complements and expands upon the findings reported by Izzo et al., who demonstrated that metformin improves mitochondrial function in DS fibroblasts primarily by restoring mitochondrial morphology, enhancing oxygen consumption rate (OCR), ATP production, and inducing the expression of mitochondrial biogenesis regulators, specifically through the activation of the PGC-1α pathway. Izzo et al. utilized metformin at concentrations of 50 µM and 500 μM, noting beneficial effects particularly at higher doses. However, it is crucial to highlight that therapeutic plasma levels of metformin typically do not exceed 30–50 μM; higher concentrations used by Izzo et al. (e.g., 500 µM) are beyond clinically relevant therapeutic thresholds, and consequently, may represent supraphysiological, potentially cytotoxic exposures (Hess et al., 2018; Dutta et al., 2023). Moreover, Izzo et al.'s research predominantly focused on the role of metformin in mitochondrial network reorganization and functional restoration via PGC-1α pathway modulation. However, it is widely recognized that metformin exerts pleiotropic effects beyond mitochondrial modulation, particularly affecting oxidative stress, inflammatory pathways, also apart from AMPK signaling (Hsu et al., 2021; Kalender et al., 2010). Our results indicate that, at therapeutically relevant concentrations (10 μM–50 µM), metformin not only modulates the activity of AMPK, PRKAA1, and NF-κB, but also significantly enhances TAC, reduces TOC, and decreases oxidative DNA/RNA damage specifically in trisomic fibroblasts. Critically, the mechanistic scope of Izzo et al.'s study is limited, focusing exclusively on mitochondrial morphology and biogenesis. It neglects the multifaceted nature of metformin’s mechanism of action—particularly its effects on systemic oxidative stress, activation of AMPK and PRKAA1, and modulation of NF-κB—as comprehensively demonstrated in our study using therapeutically relevant concentrations (10 μM–50 µM), in contrast to previous studies that employed supra-physiological doses. By addressing these additional pathways, our findings provide crucial supplementary insights, confirming that metformin’s beneficial effects in T21 cells are not exclusively mitochondria-centric but involve broader cellular signaling pathways contributing to non-AMPK pathways. This positions our study as a necessary and valuable validation to the findings of Izzo et al., addressing the incomplete nature of their mitochondrial-focused perspective and highlighting the comprehensive antioxidative and anti-inflammatory potential of metformin in T21.

Importantly, in this study oxidative stress was also confirmed at the molecular level, as evidenced by elevated concentrations of DNA/RNA OSDP in T21 fibroblasts. Notably, metformin did not alter DNA/RNA OSDP levels in normal fibroblasts (PCS-201-012), whereas in both T21 cell lines (CCL-84 and CCL-54), a consistent reduction in DNA/RNA oxidative damage was observed across all tested concentrations (10 μM, 30 μM, and 50 µM). These findings suggest that metformin exerts a direct nucleoprotective effect in trisomic cells, independent of dose variation, possibly through mechanisms not limited to AMPK activation. (Buczyńska et al., 2021a; Pietryga et al., 2021; Lim et al., 2014; Musi et al., 2002; Foretz et al., 2014; Ma et al., 2022; Zhou et al., 2001; Goel et al., 2022; Chhunchha et al., 2022; Moreau et al., 2021; Parfieniuk et al., 2018; Dierssen et al., 2020; Moreau et al., 2021; Dierssen et al., 2020; Dierssen et al., 2020; Moreau et al., 2021; Buczyńska et al., 2021a; Buczyńska et al., 2023; Buczyńska et al., 2021a; Muchová et al., 2014; Muchová et al., 2001; Strydom et al., 2018; Hasanvand, 2022; Buczyńska et al., 2021a; Schmitz et al., 2022; Rius-Pérez et al., 2020). Interestingly, the increased NF-κB activity observed in our study may contribute to exacerbating the pro-inflammatory environment typically associated with T21. Importantly, this pro-inflammatory effect appears not to be inhibited effectively by AMPK activation, suggesting that NF-κB signaling in T21 fibroblasts may involve additional regulatory mechanisms independent of, or resistant to, AMPK-mediated suppression (An et al., 2025). This observation emphasizes the complexity of NF-κB regulation in trisomic cells and highlights the necessity for further exploration into alternative signaling pathways and mechanisms that modulate responses in T21 (Wilcock and Griffin, 2013). The study performed by Salemi et al. raveled that NF-κB1 expression is reduced in T21 fibroblasts compared to those from euploid individuals, suggesting a potential role in the dysregulation of apoptotic pathways. Given that NF-κB1 is involved in cell survival, immune regulation, and inflammation, its diminished expression in T21 fibroblasts may contribute to enhanced pro-apoptotic signaling, impaired immune cell development, and delayed cellular proliferation (Salemi et al., 2015). The NF-κB subunits are not direct phosphorylation targets of AMPK, but the inhibition of NF-κB signaling is mediated by several downstream targets of AMPK, e.g., SIRT1, PGC-1α, p53, and Forkhead box O factors. In conducted research NF-κB levels decreased in the control group (PCS-201–012 line) after the administration of metformin at doses of 30 µM (p = 0.02) and 50 µM (p < 0.0001). In the experimental group, both the CCL-84 and CCL-54 lines, shows an increase in NF-κB levels following metformin treatment at doses of 30 µM (p < 0.0001) and 50 µM (p = 0.042) in line CCL-84 and 10 µM (p = 0.047) and 30 µM (p < 0.001) at CCL-54 line, compared to baseline values. The observed decrease in NF-κB levels in the control group and increase in T21 fibroblasts across various dosages suggest that metformin may have a context-dependent role in modulating inflammatory responses and origin from decreased expression of NF-κB among DS individuals (Salemi et al., 2015). In healthy cells, metformin likely activates NF-κB to support immune signaling. However, in T21, where chronic inflammation and immune dysfunction are prevalent, the increased NF-κB levels—particularly at higher doses like 50 μM—may reflect a complex interplay between elevated total oxidant capacity (TOC) and an impaired immune response. This may lead to a compensatory normalization of NF-κB levels, given that baseline concentrations were initially decreased compared to euploid controls (Huggard et al., 2020; Malle et al., 2023). Rather than being a pathological response, this activation may represent an attempt to overcome the immune deficiencies observed in T21 (Sutter et al., 2024). This suggests that metformin could contribute to rebalancing the immune response, supporting an adaptive mechanism to mitigate systemic inflammation and oxidative stress in T21 (Sutter et al., 2024; Busciglio et al., 2002; Wilcock and Griffin, 2013; Sukreet et al., 2024; Eun et al., 2008). Importantly, our results align with the studies conducted by Moiseeva et al., which demonstrated that in fibroblasts derived from individuals with T21, metformin application increases NF-κB levels through a multifaceted process involving IκB kinase (IKK) inhibition, cytokine modulation, and AMPK activation. The inhibition of IKK prevents the degradation of IκB proteins, which in turn regulates NF-κB activation, preventing excessive inflammatory responses despite elevated NF-κB level (Moiseeva et al., 2013). Furthermore, insulin resistance can trigger inflammatory pathways, elevating NF-κB activity and increasing cellular stress (Shoelson et al., 2006). Metformin’s anti-inflammatory properties suggest its role in managing chronic inflammation associated with metabolic disorders. By targeting multiple signaling pathways, including AMPK activation, mTOR inhibition, and modulation of JAK/STAT and IL-6 pathways, metformin demonstrates potential in addressing both metabolic and inflammatory dysregulation (Lin et al., 2023; Saleiro and Platanias, 2015; Amin et al., 2019). Future studies should explore whether metformin selectively enhances the regulatory functions of NF-κB in T21, potentially mitigating apoptosis while avoiding excessive pro-inflammatory activation. A detailed transcriptomic or proteomic analysis of NF-κB target genes in response to metformin treatment could provide further insights into these mechanisms.

While this study provides insights into the potential effects of metformin in T21, several limitations must be acknowledged. The *in vitro* design using commercially available fibroblast lines may not fully reflect the complexity of T21-related metabolic dysfunction *in vivo*. Additionally, while metformin influenced oxidative stress markers and key pathways (AMPK, PRKAA1 and NFkB), the study does not establish a direct mechanistic link to mitochondrial outcomes. Further metabolomic and transcriptomic analyses, along with *in vivo* validation, concerned on mitochondrial activity assessment are needed to confirm these findings. Given these limitations, the results should be considering as preliminary, and future studies should focus on refining therapeutic strategies for T21. The measurements represent absolute concentrations as obtained from commercial ELISA kits and were not normalized to total protein content. Moreover, given the preliminary nature of these findings, further validation through Western blotting and gene expression profiling is warranted. Nevertheless, to our knowledge, this is one of the few studies that systematically evaluates how metformin influences redox status and key inflammatory and metabolic signaling pathways specifically in trisomic cells on clinically commonly used doses.

The utilization of metformin in pregnancy is gaining significant traction worldwide, bolstered by an expanding array of evidence from randomized controlled trials (RCTs) that affirm its safety and effectiveness (Buczyńska et al., 2022; Tso et al., 2020; Al-Ruthia et al., 2017; Ghomian et al., 2019). Recent investigations have shown that metformin can achieve pregnancy outcomes comparable to those obtained with insulin therapy, while also contributing to reduced maternal weight gain and high levels of patient satisfaction. Presently, a multicenter RCT is being conducted to assess the effects of adding metformin to insulin therapy for pregnant women diagnosed with type 2 diabetes (Wu et al., 2020; Newman and Dunne, 2022; Morin-Papunen et al., 2012; Vause et al., 2010). Up to date, Pashou et al. highlight metformin’s efficacy and safety during pregnancy, noting its ability to reduce insulin requirements and decrease the incidence of complications such as hypertensive disorders. The review indicates that metformin’s mechanisms—including enhanced insulin sensitivity and reduced hepatic glucose production—significantly increase its therapeutic effectiveness (Paschou et al., 2024). Additionally, studies by Skibinska et al. demonstrate that metformin administration effectively reduces the incidence of gestational diabetes mellitus (GDM) and associated complications, including hypoglycemia and respiratory distress syndrome, thereby supporting improved maternal and neonatal health outcomes (Skibinska et al., 2021). In following examination of metformin, Newman et al. analyze its effectiveness in managing diabetes during pregnancy, focusing on both its benefits and potential drawbacks. Metformin is noted for its proven efficacy in glycemic control and its ability to reduce the risk of macrosomia, a condition that can lead to complications during delivery, such as preterm birth, hypertensive disorders, and higher cesarean delivery rates (Newman and Dunne, 2022). Gastrointestinal side effects associated with metformin treatment can impact patient adherence to the regimen. However, evaluations of metformin’s effects from preconception through breastfeeding reveal that children exposed to metformin *in utero* experience favorable metabolic outcomes, including a reduced risk of obesity and type 2 diabetes (Newman and Dunne, 2022; Tosti et al., 2023). Nevertheless, the increasing prevalence of diabetes during pregnancy has prompted extensive research into effective management strategies. The MiTy trials confirm that metformin is a viable option for women with type 2 diabetes during pregnancy, demonstrating significant improvements in glycemic control compared to placebo (Feig et al., 2020). Feig et al. (2020) Furthermore, the MOMPOD trials support the use of metformin in combination with insulin for managing preexisting diabetes and GDM in early pregnancy, highlighting its significant role in improving maternal health outcomes (Boggess et al., 2023). Future clinical studies should extend their scope beyond GDM to explore metformin’s potential in addressing the metabolic challenges of pregnancies affected by T21, which is increasingly recognized as a metabolic syndrome (Moreau et al., 2021; Parfieniuk et al., 2018; Dierssen et al., 2020; Yahia et al., 2021). This area demands in-depth investigation, as understanding the metabolic alterations associated with T21 could pave the way for more targeted and effective therapeutic strategies. Furthermore, metformin’s therapeutic potential should not be limited to pregnancy. Its application across the lifespan, including in children, adolescents, and adults with T21, warrants exploration given its demonstrated ability to modulate oxidative stress, inflammation, and metabolic pathways. Future research should prioritize optimizing dosing regimens, evaluating long-term effects, and identifying predictive biomarkers of therapeutic efficacy to maximize metformin’s benefits.

A deeper understanding of metformin’s mechanisms in T21 could revolutionize treatment approaches, enhancing metabolic health and improving quality of life for individuals with T21 at all stages of development. This highlights metformin’s potential as a versatile and impactful intervention in managing the complexities of T21.

## 5 Conclusion

Our study provides evidence that metformin significantly reduces oxidative stress and enhances antioxidant capacity in T21 fibroblast cell lines, as demonstrated by TOC and DNA/RNA OSDP, along with increased TAC. Additionally, metformin modulates key signaling pathways, including AMPK and PRKAA1 activation and an increase in NF-κB levels, particularly in T21 cells. These findings suggest that metformin plays a dual role in mitigating oxidative imbalance and modulating inflammatory responses, supporting its potential therapeutic application in T21. Future research should focus on determining optimal dosing strategies, evaluating long-term safety, and identifying predictive biomarkers to refine therapeutic efficacy. A deeper understanding of metformin’s molecular mechanisms in T21 could pave the way for targeted interventions, ultimately improving health outcomes and quality of life for individuals with T21.

## Data Availability

The raw data supporting the conclusions of this article will be made available by the authors, without undue reservation.
